# Differential Protein Expression in Response to Abiotic Stress in Two Potato Species: *Solanum commersonii* Dun and *Solanum tuberosum* L.

**DOI:** 10.3390/ijms14034912

**Published:** 2013-03-01

**Authors:** Raquel Folgado, Bart Panis, Kjell Sergeant, Jenny Renaut, Rony Swennen, Jean-Francois Hausman

**Affiliations:** 1 Department Environment and Agro-biotechnologies (EVA), Centre de Recherche Public—Gabriel Lippmann, 41, rue du Brill, L-4422 Belvaux, Luxembourg; E-Mails: folgado@lippmann.lu (R.F.); sergeant@lippmann.lu (K.S.); renaut@lippmann.lu (J.R.); 2 Laboratory of Tropical Crop Improvement, Division of Crop Biotechnics, KU Leuven, Willem de Croylaan, 42 bus 2455, B-3001 Leuven, Belgium; E-Mails: bart.panis@biw.kuleuven.be (B.P.); rony.swennen@biw.kuleuven.be (R.S.); 3 Bioversity International, Willem de Croylaan, 42 bus 2455, B-3001 Leuven, Belgium

**Keywords:** abiotic stress, osmotic stress, cold acclimation, potato, proteomics, proteinase inhibitors

## Abstract

Better knowledge on responses to dehydration stress could help to improve the existing cryopreservation protocols for potato, since plant tissues processed for cryopreservation are often submitted to similar *in vitro* stress conditions. Cryopreservation (the best method of conservation for vegetatively propagated plants) of potato still needs to be standardized to make it available and to conserve the wide diversity of this crop. In the present work, the response to osmotic stress and chilling temperature was investigated in two potato species, *Solanum tuberosum* and its relative, frost-tolerant *S. commersonii*. After 14 days of exposure, different growth parameters, such as shoot length and number of leaves, were measured. Furthermore, differentially abundant proteins were identified after performing 2-fluorescence difference gel electrophoresis (2-DIGE) experiments, and soluble carbohydrates were analyzed by High Performance Anion Exchange Chromatography with Pulsed Amperometric Detection (HPAEC-PAD). The results show different responses in both species depending on the stress treatment. Focusing on the differences in growth parameters during the treatments, *Solanum commersonii* seems to be more affected than *S. tuberosum* cv. Désirée. At the molecular level, there are some differences and similarities between the two potato species studied that are dependent on the type of stressor.

## 1. Introduction

Potato (*Solanum* spp.) is one of the most cultivated species on earth and ranks fifth in production [1]. This tuber-bearing crop is not only rich in carbohydrates, but it is also a source of minerals and vitamins. In the developing world, potato is present in the diet of half a billion consumers [2]. Potato is known to have the richest genetic diversity of all cultivated plants [3]. However, the improved commercial varieties consumed around the world have mainly been generated from only one of the eight existing species, *i.e.*, *S. tuberosum* subsp. *tuberosum*.

Environmental constraints, like drought or low temperatures, can have adverse effects on plants and, as a consequence, can induce stress responses at the molecular and physiological level [4] that often leads to yield loss. Furthermore, if such exposure is extended in time, stress causes major alterations in the plant metabolism that ultimately leads to cell death [5]. Most of the crop plants necessitate optimal growth conditions to maintain a stable production. However, despite extensive research and the use of sophisticated and intensive crop-protection measures, losses due to dehydration stress amount to the billions of dollars annually.

For potato, frost is the abiotic constraint that causes the most severe yield losses. However, there are several potato species, such as *S. commersonii*, that are able to cold acclimate and increase their frost tolerance [6]. Although *S. commersonii* does not have a commercial value, this species has been studied for its resistance traits. The conservation of this and other potato species with low commercial value is thus important. The best method to date for the long-term conservation of vegetatively propagated plants is cryopreservation. Indeed, during cryopreservation, cell division, metabolic and biochemical processes are arrested, and thus, the plant material can be stored for a long period of time [7].

An important step in many cryopreservation protocols is the exposure of *in vitro* cultivated plants to stresses similar to drought, salinity and frost prior to the cryopreservation. Therefore, it is expected that studies on the mechanisms by which plants respond to dehydration stresses can be used for the improvement of cryopreservation methods, currently with often low and unpredictable results [8]. Moreover, one of the most common acclimation mechanisms related to freezing tolerance is the accumulation of osmo-active compounds, a mechanism that is induced by drought and cold exposure. Soluble sugars form an important part of these osmo-active molecules and are furthermore involved in various metabolic events and can act as signal molecules regulating gene expression and protein activity, especially those involved in photosynthesis, sucrose metabolism and osmolyte synthesis [9].

Transcriptome studies using microarray technology have identified several genes that are overexpressed during dehydration and rehydration [10–13]. However, several studies have shown that changes in mRNA transcript levels do not automatically imply corresponding changes in protein amount or activity [14,15]. A proteomic approach complements the genomic and transcriptomic data by looking at the actual protein population of a specific tissue, cell or cellular compartment. Proteomic approaches aim at analyzing the complex reactions of plants and are aided by sensitive and rapid protein identification that relies on mass spectrometry [16]. Proteomic studies of plant response to dehydration stress include analyses of the effects of water deficit, salt excess, low and high temperatures, high light and the presence of toxic chemicals, such as herbicides or heavy metals in the environment [17]. Several studies have shown that a combination of proteomic exploration with metabolomic and genetic approaches allows a better integrated understanding of plant responses to dehydration [18,19]. Several proteome studies have mainly been executed on tuber tissue [20–22] and in connection with phytopathogens [23–25]. Other studies on protein changes related to abiotic stress tolerance in potato have also been implemented until now [15,26].

The present work includes a proteomic study on the effects of the different treatments and the effects of these on *in vitro* shoots of two potato species. Moreover, we analyzed the amounts of soluble sugars that, combined with the proteomics approach, show some of the metabolites that undergo changes under different preculture conditions. Furthermore, to our knowledge, this is the first time that this type of combined study (proteomic and targeted metabolites analyses) compares *S. tuberosum* with its frost-tolerant relative, *S. commersonii*, to highlight some common and different mechanisms in response to abiotic stress.

## 2. Results and Discussion

Potato is considered as a model crop for *in vitro* culture techniques [27]. Moreover, it has been shown that *in vitro* culture can be a good system to screen for salinity [28] and drought tolerance [29,30] in potato. Monitoring the molecular events underlying this tolerance provides important information for breeding programs. Osmotic active compounds, such as sorbitol and mannitol, have been used as *in vitro* stress agents in order to select drought-tolerant genotypes in different crops. Sucrose is known to be a key osmolyte and metabolite, which is involved in drought, as well as cold, tolerance. Sucrose is the major sugar transported in plants, and it plays also an important role as a signaling molecule in regulating gene expression and plant development [31,32].

Prior to the present study, we performed a preliminary experiment to define the optimal experimental conditions (results not shown). For this, we tested different concentrations of three sugars (sucrose, sorbitol and mannitol) for three weeks. Sorbitol and mannitol seemed to have a similar effect when we compared them, so we selected sucrose and sorbitol as osmotic stressors. Chilling temperatures (temperature below optimal, but above freezing) above 4 °C are often used as preculture to cold acclimate the *in vitro* plants before submitting them to cryo-procedure; we decided to use 6 °C as the chilling temperature, because it negatively affects growth.

### 2.1. Morphological Measurements

**Figure 1:**
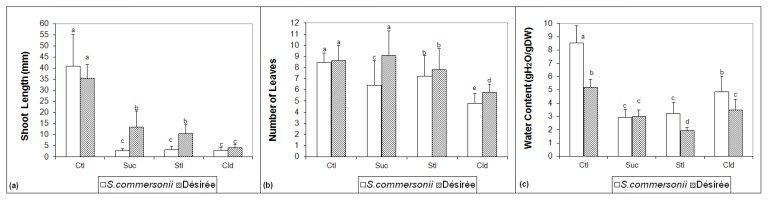
Three-week-old *in vitro* shoots of two potato species (*S. commersonii* and *S. tuberosum* L. cv. Désirée) submitted to the different stress treatments for two weeks. Three parameters are shown: (**a**) shoot length, (**b**) number of leaves and (**c**) water content. Letters represent statistical significance for ANOVA *p* < 0.05. Error bars represent standard deviation. Ctl = control; Suc = sucrose; Stl = Sorbitol; Cld = Cold.

The phenotypical study of the two potato species used for this study after two weeks of treatment reveals that the growth rate is compromised by all three treatments used (Figure 1).

The shoot length is significantly lower in *S. commersonii* when plants are exposed to a high sugar concentration compared to control conditions (Figure 1a). Moreover, *S. commersonii* plantlets show the same increase of shoot length independent of the sugar used, whereas, in Désirée, sucrose affects shoot length less than sorbitol. There is no significant difference in shoot length when the plants are grown at chilling temperature.

The number of leaves is significantly lower in the plantlets submitted to stress conditions when compared to the controls, except for Désirée plantlets submitted to sucrose (Figure 1b). The effect of sorbitol is similar in both species. Sucrose and cold affect the number of leaves more in *S. commersonii*.

The water content is significantly lower in both species for the three constraints (Figure 1c) when compared to control. For *S. commersonii*, sucrose and sorbitol treatments induce more reduction of water content than cold. For Désirée, there are no significant differences in the effect on this parameter between sucrose and cold treatments.

### 2.2. Soluble Sugars Accumulation

**Figure 2:**
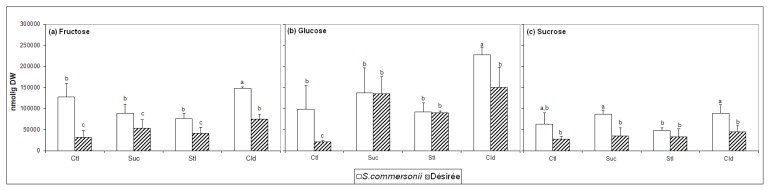
Quantification of (**a**) glucose, (**b**) fructose and (**c**) sucrose. Statistical significance was tested by one-way ANOVA between control and treated and between one species against the other of the same condition. Letters represent statistical significance for ANOVA *p* < 0.05. *n* = 5. Error bars represent standard deviation. Abbreviations: Ctl, Control; Suc, Sucrose; Stl, Sorbitol.

The measured levels of the main carbohydrates (fructose, glucose, sucrose) are represented in Figure 2. The concentration of fructose, glucose and sucrose is higher in *S. commersonii* compared to Désirée. These three metabolites increase significantly in concentration under cold treatment in *S. commersonii* as compared to their control. Fructose content is stable in plants exposed to osmotic stress. Sucrose concentration is significantly higher in plants of *S. commersonii* treated with sucrose. Glucose content increased in Désirée plants when treated with sucrose and sorbitol.

Other carbohydrates were detected in this study, such as galactose, melibiose, raffinose and stachyose. The data are presented in Table S1.

### 2.3. Proteome Study

In order to determine the proteome-level differences between the two potato species grown under control conditions, a one-way ANOVA was performed on the gel maps from the control plants. The effects of abiotic stresses were tested with a two-way ANOVA, with species as factor one and treatment as factor two.

**Figure 3:**
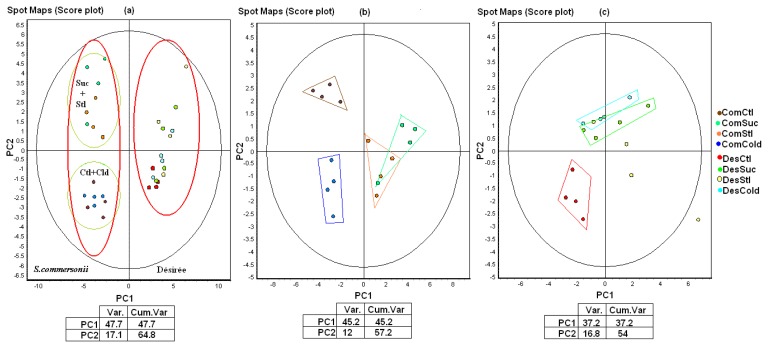
Principal component analysis based on the variability between gels of the two potato species. (**a**) *S. commersonii* and *S. tuberosum* Désirée; (**b**) *S. commersonii*; (**c**) *S. tuberosum* Désirée. Abbreviations: Com, *S. commersonii*; Des, *S. tuberosum* Désirée; Ctl, Control; Suc, Sucrose; Stl, Sorbitol; Var., variance; Cum. Var, cumulative variance.

The distribution of the spot maps on the principal component analysis (PCA) plot given in Figure 3a shows that the most important differences were found between the samples of *S. commersonii* and Désirée (Figure 3a; the X axis differentiates the species), irrespective of the treatment. In order to better consider differences among different treatments, the spot maps from each species were processed separately. PCA analysis of the spot maps from *S. commersonii* resulted in the separation of three groups: Control, Cold and Sucrose plus Sorbitol (Figure 3b). The spot maps corresponding to Désirée can be separated into two clear groups: control and the stress treatments (Figure 3c).

Differentially abundant proteins with a ratio of ≤1.5- or >1.5-fold and a *p*-value < 0.05 were selected. In total, this resulted in the selection of 310 spots that were significantly different; of these, 283 spots could be reliably matched and were finally picked on the selected gel, as described. In 210 of these spots, a protein was significantly identified. Elimination of spots from which two or more proteins were identified resulted in a final list of 94 spots/proteins that were considered in this study. When two or more proteins are identified in the same spot, it means that different co-migrating proteins are present. Because when using the normal procedures for two-dimensional polyacrylamide gel electrophoresis (2D-PAGE) there is no way to differentiate which of two co-migrating proteins is changing in abundance, identifying a spot as being significantly different when plants are treated *versus* untreated means nothing if a second protein is also present in this spot.

**Figure 4:**
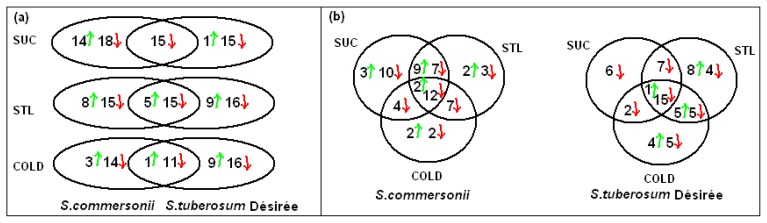
Venn diagram representation of the proteomic results of the sucrose, sorbitol and cold exposure datasets. (**a**) Comparison between *S. commersonii* and *S. tuberosum* Désirée; (**b**) Comparison among the three constrains (sucrose, sorbitol and cold) in the same species. The numbers represent the number of identified proteins that are significantly increased (with green arrows) or decreased (with red arrows) in abundance (*p* value < 0.05) in *S. commersonii* and *S. tuberosum* cv. Désirée. Abbreviations: Suc, Sucrose; Stl, Sorbitol.

**Figure 5:**
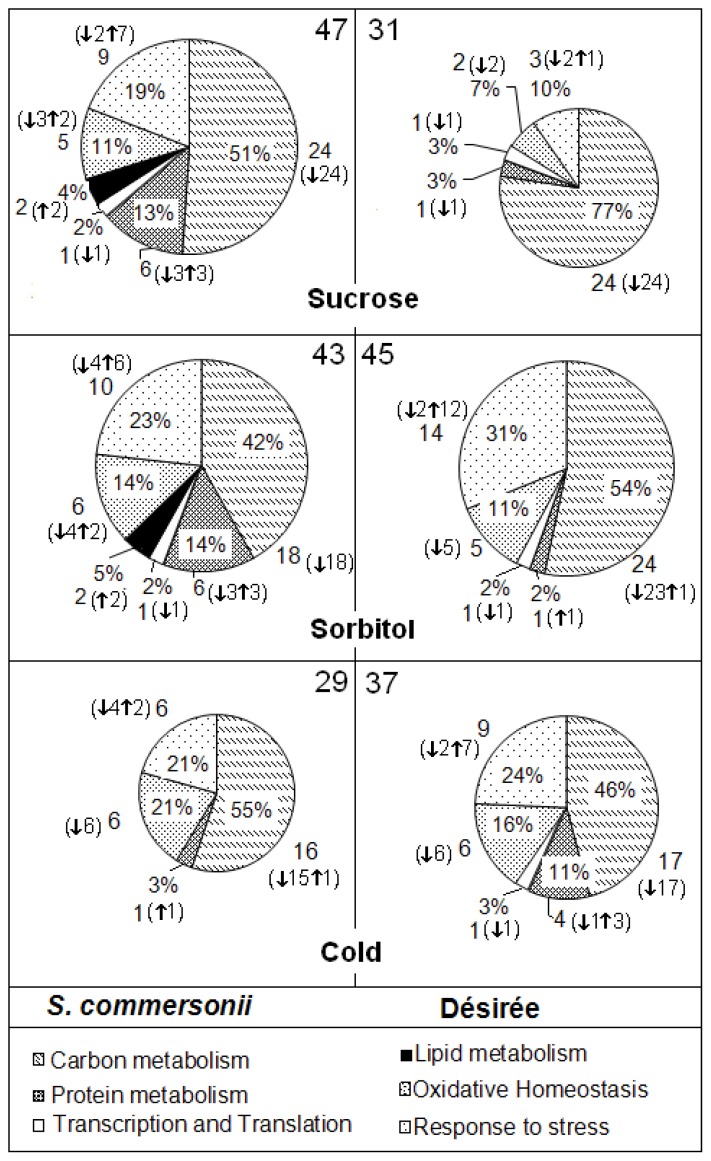
Distribution of identified proteins differentially expressed under the different stress conditions studied. The numbers represent the number of identified proteins that significantly change in abundance in *S. commersonii* (**left**) and *S. tuberosum* cv. Désirée (**right**); between brackets are represented the number of proteins that are significantly increased (↑) or decreased (↓) in abundance. Classification of proteins was done based on the GO annotation from Goanna [33].

The proteins discussed below are presented in Table 1. From a Venn diagram representation, we can see how many spots are common when comparing the treatments in the same species (Figure 4a) and also how many spots are common when comparing both species in the same treatment (Figure 4b). This made a final total of 47 identified spots for *S. commersonii* sucrose (14 up and 33 down), 31 for Désirée sucrose (one up and 30 down), 43 for *S. commersonii* sorbitol (13 up and 30 down), 45 for Désirée sorbitol (14 up and 31 down), 29 for *S. commersonii* cold (four up and 25 down) and 37 for Désirée cold (10 up and 27 down) (Figure 5).

#### 2.3.1. Differences between Species (Control Conditions)

*Solanum commersonii* is known to be the most frost tolerant species of potato together with *Solanum acaule* [34,35]. Cultivated potato, *Solanum tuberosum*, is sensitive to freezing [36] and possesses little genetic variation in their tolerance to cold stress [6,37]. Furthermore, *S. commersonii* chloroplast DNA has been shown to be different from other frost sensitive species [38], and it has been suggested that such differences might be linked with frost hardiness.

In the present work, from the 94 identified proteins, 48 appear to be differentially expressed when we compared *S. commersonii* control to Désirée control (Table 1). Among those, 32 spots are more abundant in *S. commersonii*, and 13 of those spots contain ribulose-1,5-bisphosphate carboxylase oxygenase (RuBisCO). Although there is no evidence that the RuBisCO activity is different in *S. commersonii* and *S. tuberosum*, RuBisCO from *S. commersonii* has been shown to be more stable during freeze-thaw cycles than *S. tuberosum* [39]. We observe that nine spots that contain RuBisCO decrease in abundance when Désirée plants are submitted to chilling temperatures, whereas, in *S. commersonii*, their change is not significant, except for one spot that increases in abundance.

#### 2.3.2. Common Changes to All Treatments in Both Species

At the proteomic level, many photosynthesis-related proteins are differentially expressed in response to both osmotic stress and cold (Table 1). All of them show a significant decreased amount, among them, PS I and PS II subunit proteins and ferredoxin reductases. Moreover, the decrease in abundance of PSII oxygen-evolving complex was identified in Désirée, and the decrease in abundance of the Oxygen-evolving enhancer protein in *S. commersonii*. This indicates that the system is responding to reduce the electron transport. Cell growth is indeed among the primary processes to be affected by abiotic constraints, especially drought [40,41] and cold [42,43].

We found other differentially expressed proteins, such as annexin p34 and germin-like protein; both of them are of lower abundance in all the treatments and in both species. The annexins have different functions, among them an actin-binding, nucleotide phosphodiesterase activity that can be abolished by Ca^2+^-dependent interaction with phospholipids [44]. A decrease of this protein might affect the properties of the cytoskeleton and cellular membrane. The germins have been shown to be associated with various aspects of plant development [45,46], such as the defense system [47,48], embryonic development, photoperiodic oscillations [49] and hormonal stimuli [50]. One of the final effects of their activity is related to cell wall expansion [49] and affects cell growth. Thirteen rice germin-like proteins are induced by abiotic stresses, including drought, cold and salt [51]. In the case of water deficit, some studies showed that the expression of germins is downregulated during the constraint and that the content of some germins increased after rewatering [52,53].

Although they do not appear as a homogeneous response, we identified some other differentially expressed proteins in all of the studied conditions. Such is the case of the different peroxidases; they generally decrease, but there are some spots containing peroxidase isoforms that increase in intensity in *S. commersonii* when growing when exposed to osmotic stress. When blasting the peroxidase sequences found in the different spots, two differentiated groups were found; one of them with the highest homology to peroxidase 12 proteins from other species (spots 746 and 577) and another that could not be linked to a specific group of peroxidases (spots 601 and 703) (Table S3).

The protein function most frequently identified among the differentially expressed spots is that of proteinase inhibitor. Numerous spots containing truncated sequences of this protein were found, but the biological significance of the observed cleavage events is unclear (Table S3). The clustering of the identified sequences reveals that the members of groups of proteinase inhibitors differentially accumulate during exposure to the treatments applied. The majority of those that increase in intensity were observed in plants exposed to osmotic stress (Table S4). The multitude of genes coding for the major proteinase inhibitors is known [54]. Plant proteinase inhibitor proteins have been shown to have functions in various physiological and developmental processes [55]. Reports on proteinase inhibitor II (PIN2), a serine-proteinase inhibitor, which occurs in Solanaceae, show that it could play a role in environmental responses and development [56,57]. Proteinase inhibitor proteins of the Kunitz family were identified from salt-treated radish [58] and drought-stressed *Arabidopsis thaliana* [59]. Also, some proteinase inhibitor genes were identified by Legay *et al.* [60] in drought-stressed advanced clones of potato. Protease inhibitor induction during drought exposure or wounding is suggested to be part of a general mechanism of damage prevention against proteases that are activated by protein denaturation due to osmotic stress [61]. In native potato, proteinase inhibitor genes were rather induced in a drought susceptible clone than a tolerant clone, suggesting that the upregulation of these genes is more associated with a stress than a tolerance response [62]. Proteinase inhibitors could be acting as a signaling system and also as a protecting system. This, however, cannot be confirmed from our results, as we do not have enough information about the different function associated to different families. The majority of proteinase inhibitor proteins identified in the present work increased in abundance under osmotic stress induced by sorbitol. However, under high sucrose concentration and also under chilling temperatures, the response is distinct when comparing both species. In the case of the sucrose treatment, six spots containing proteinase inhibitors increase in abundance in *S. commersonii*, whereas there is only one in the case of Désirée. Under chilling temperatures, proteinase inhibitors in Désirée increase, and in *S. commersonii*, three of the spots are less abundant than in control conditions; only one increased in abundance. This suggests that at chilling temperature, a cold tolerant ecotype is not significantly affected by the stress application, so that it does not need a very important system of protection, as proteinase inhibitors.

The results suggest that a basal response to both osmotic and cold stressors is mainly associated with C metabolism. However, we did not find a common pattern among the soluble carbohydrates measured (Figure 2), which might be attributed to interactions with other pathways that converge with these different sugars.

#### 2.3.3. Specific Responses during Osmotic Treatments

Apart from the proteins that change in quantity in all the studied conditions, other proteins vary their abundance only under osmotic stress. We found some proteins that decrease in abundance only in Désirée, like triose phosphate isomerase and cytosolic ascorbate peroxidase. Triose phosphate isomerase has been found to decrease under stress conditions, such as ozone [63] and drought [64]. In the processes of glycolytic synthesis of ATP, triose phosphate isomerase catalyzed the isomerization of dihydroxyacetone phosphate to glyceraldehyde 3-phosphate. The decrease in abundance of this and other proteins could lead to an inhibition of glycolysis and the tricarboxylic acid cycle and, consequently, arrest mitochondrial metabolism under oxidative stress conditions, leading to the prevention of the deleterious production of reactive oxygen species [65]. Cytosolic ascorbate peroxidase activity is essential for the oxidative protection of chloroplasts in plants subjected to abiotic stresses [66–70]. Although it has been reported that the loss of the cytosolic ascorbate peroxidase function in plants resulted in lower photosynthetic rates, slower growth and delayed flowering under normal growth conditions [68,71,72], the role of cytosolic ascorbate peroxidase in plant abiotic stress responses has not yet been elucidated [73,74].

On the other hand, in *S. commersonii* exposed to osmotic stress, spots containing proteins, like chaperonin 21, 30S ribosomal protein S5 family protein and chlorophyll a-b binding 8, decrease in intensity, while spots containing polyubiquitin 2 increase. These proteins are all involved in protein synthesis and degradation, are located in the chloroplast and have been reported to be affected by abiotic stress [75–78]. The molecular chaperones that belong to the chaperonin family are essential components that are required for the folding of proteins within cells. Chaperonin 21 has been reported to act as co-chaperonin, helping chaperonin 60, whose function is to facilitate the assembly of RuBisCO [79,80]. The 30S ribosomal protein S5 family is one of the plastid-specific ribosomal proteins, directly implicated in the translation of RNA into the plastids. Chloroplast transcription-translation has evolved numerous additional control elements to achieve its highly effective coordination between photosynthetic protein requirements and the ribosome function [81]. Proteins of this translation machinery are integral components of the adaptation to desiccation stress, as the enhancement of the translation machinery would allow for expression of cellular stress response proteins under dehydration, where rates of protein production significantly and progressively decline [76]. Protein degradation is important in the reorganization of plant metabolism under stress [82]. The ubiquitins have been shown to have a role in the turnover of proteins in chloroplast, including photosynthesis-and carbon fixation-related proteins [83,84]. Our results indicate that plastid-specific protein synthesis is inhibited, and degradation is facilitated under osmotic stress condition in *S. commersonii* and suggests a more pronounced stress-related response in this species, as compared to Désirée.

We also found several spots containing patatin that increase in *S. commersonii* under osmotic conditions. Patatins are a group of plant storage glycoproteins that show lipid acyl hydrolase activity. The patatin-associated lipolytic activity was thought to be a means of defense against plant parasites and has been shown to function in plant signal transduction as well [85,86].

These results, together with the measured carbohydrates, suggest specific responses at the molecular level for each species that are moreover observed at the morphological level.

#### 2.3.4. Changes Produced by Cold Treatment

*S. commersonii* is known to be the most frost hardy wild potato species, being able to tolerate an acute freezing episode of about −5 °C and further acclimate to tolerate −10 °C after being exposed to chilling temperatures for several days [6,87]. On the other hand, *Solanum tuberosum* cv. Désirée is only slightly able to acclimate to cold, and it is thus rather sensitive to frost [26].

The number of differentially expressed proteins is higher in Désirée than in *S. commersonii* in cold treatment, which suggests that more metabolic changes might occur in Désirée

We have identified a heat-shock protein 70 (Hsp70) that increases its quantity in *S. commersonii* after exposure to chilling temperature. Hsp70 has been shown to prevent cytochrome *c*/dATP-mediated caspase activation and recruitment of caspases to the apoptosome complex. Hsp70, therefore, suppresses apoptosis [88]. This protein also increases significantly in abundance in Désirée sucrose and cold treatment, but to a lower extent. Another heat-shock protein, putative RuBisCO binding-protein alpha subunit, was specifically more abundant in Désirée.

We identified a pathogenesis-related 10 (PR10) protein that also increased in abundance in Désirée after cold exposure. Evers *et al.* [15] showed the upregulation of the PR10 gene and an increased accumulation of the PR10 protein in the same Désirée cultivar under chilling temperature.

The accumulation of simple sugars is a common response for both species. Fructose and glucose concentration increased after cold exposure. We also found an increase of galactose in Désirée, but not in *S. commersonii* (Table S1). This suggests a divergence in some pathways as a response to cold temperatures that might lead to a different cold acclimation process.

## 3. Experimental Section

### 3.1. Plant Material and Stress Treatments

Vegetatively-propagated apical potato shoots from 3-week-old *in vitro* plantlets of two varieties of potato (the commercial cultivar *S. tuberosum* L. cv. Désirée and the wild potato *S. commersonii* spp. *commersonii*, provided by the CGR, the Netherlands) were submitted for 2 weeks to osmotic stress at 22 °C, 16/8 h day/night and a light intensity of 40 μmol m^−2^ s^−1^. The Murashige and Skoog (MS) medium (MS salts and vitamins + 0.09 M sucrose) was taken as control [89], while the osmotic treatment was provided by MS medium supplemented with 0.21 M of sucrose and MS medium plus 0.21 M of sorbitol (resulting in a total sugar concentration of 0.3 M). A fourth batch of plantlets was cultivated in MS medium, but at 6 °C in order to evaluate the changes occurring in the plant tissues after exposure to chilling temperatures.

After the treatments, shoots from *in vitro* plantlets were collected and stored at −80 °C. Five biological replicates per treatment, composed of a pool of 12 shoots grown in the same culture container, were sampled for both protein and carbohydrate analyses.

### 3.2. Morphological Study

After 14 days of exposure to all the conditions, different growth parameters were recorded: length of shoot, number of leaves, length and number of roots. Additionally, fresh and dry weights were measured in order to calculate the water content of the shoots.

Data presented (Figure 1) are the means of values of at least three independent experiments, with 12 explants per treatment. Data were analyzed by using one-way ANOVA, and comparisons among the mean values were evaluated by the Turkey’s multiple range test at *p* < 0.05.

### 3.3. Soluble Protein Extraction and Labeling

Proteins were extracted from samples that were composed of six to eight shoots grown in the same culture container; five samples were taken per treatment and per species, amounting to five biological replicates.

For the extraction of soluble proteins, shoots were crushed in liquid nitrogen and mixed with 10 mL 20% trichloroacetic acid (TCA) and 0.1% *w*/*v* dithiothreitol (DTT) in ice-cold acetone and kept overnight at −20 °C. After centrifugation for 45 min at 30,000× *g* and 4 °C, the pellets were washed twice with ice-cold acetone before drying. Dried samples were re-suspended in labeling buffer (7M urea, 2 M thiourea, 4% *w*/*v* 3-[(3-Cholamidopropil) dimethylammonio]-1-propanesulfonate (CHAPS), 30 mM Tris) and incubated for 1 h at room temperature. After centrifugation (15,000× *g*, 15 min), the supernatant was transferred to 1.5 mL tubes.

Prior to quantification using the 2D Quant Kit (GE Healthcare; Little Chalfont, UK) with bovine serum albumin (BSA) as standard, the pH of the protein extracts was adjusted to about 8.5.

Four of five biological replicates were used for analysis by electrophoresis.

### 3.4. Electrophoresis

After extraction, the soluble proteins were used for analysis by fluorescence difference in gel electrophoresis (DIGE) [90].

The samples were homogenized by vortexing and centrifuged. Prior to electrophoresis, all protein extracts and a pooled internal standard were labeled with CyDyes™ (GE Healthcare), according to Kieffer *et al.* [78]. A volume, equivalent to 30 μg of protein, of each protein sample was labeled with 240 pmol of the dyes. Ninety micrograms of proteins (2 samples of 30 μg each and 30 μg of internal standard), the volume adjusted to 120 μL with lysis buffer (labeling buffer without Tris and with bromophenol blue) and 2.4 μL of Destreak Reagent (GE Healthcare) and 0.72 μL of IPG buffer 3–10 NL (GE Healthcare) were added and then loaded by cup loading on 24 cm 3–10 NL ReadyStrip™ IPG strips (Bio-Rad Laboratories, Inc, Hercules, CA, USA). Prior to the cup loading, the ReadyStrip™ IPG strips were rehydrated overnight with 450 μL of rehydration solution (prepared with 500 μL of Destreak rehydration solution (GE Healthcare) and 5 μL of IPG buffer 3–10 NL (GE Healthcare) [58].

Isoelectric focusing (IEF) was carried out on an Ettan IPGphor Manifold (GE Healthcare) in an IPGphor unit (GE-Healthcare) with the following protocol: 300 V for 2 h, gradient to 1000 V over 2 h, 1000 V for 2 h, gradient to 2000 V over 2 h, 2000 V for 2 h, gradient to 4000 V over 4 h, 4000 V for 2 h, gradient to 8000 V over 4 h, 8000 V, until ~120,000 V h were reached at 20 °C, with a maximum current setting of 50 μA per strip. After equilibration, reduction and alkylation of cysteines using DTT and IAA, respectively, the strips were placed on 12.5% acrylamide precasted gels (Serva Electrophoresis, Heidelberg, Germany). The SDS-PAGE step was performed in an Ettan DALT Twelve separation unit (GE Healthcare).

### 3.5. Image Capture and Analysis

The gel images of the different samples were acquired using a Typhoon Variable Mode Imager 9400 (GE Healthcare) at a resolution of 100 μm, according to the instructions provided for each dye. Images were analyzed using the Decyder version 6.5 software (GE Healthcare). A two-way ANOVA, with species as one factor and treatment as the second factor, was performed. All spots with a significant score for one of the factors or for the interaction between the two factors (*p*-value < 0.05) were considered as spots of interest, and a total of 283 spots were submitted to MS-based identification. The ratio between control and treated spots was calculated as follows. Ratio treated/control is the ratio of the normalized log-intensities of each spot. However, to allow straightforward comparison of the fold changes, two different calculation modes were used. When the ratio is bigger than 1, the fold change is equal to this ratio. In the case that the ratio is smaller than 1 or the spot is of lower intensity in the treated samples, then the fold change is (−1/ratio).

### 3.6. Protein Identification

Spots of interest, selected as described earlier, were excised from a non-charged gel containing 90 μg of protein and digested using the fully automated Ettan Spot Handling Workstation (GE Healthcare), as described previously [91]. All MS and MS/MS analyses were performed using a 4800 MALDI TOF/TOF (Applied Biosystems, Foster City, CA, USA), and the resulting spectra used in database searches with an Applied Biosystems GPS-server and an in-house MASCOT platform (Matrix Science, London, UK). All proteins from the taxonomy Viridiplantae were downloaded from the NCBI server and used as protein database; likewise, the used EST database contained all Viridiplantae ESTs downloaded on 04/10/2010. All searches (combined MS and 8 MS/MS spectra) were carried out using a mass window of 100 ppm for the precursor and 0.75 Da for the fragments. During the different searches, the following parameters were defined: two missed cleavages, fixed carbamidomethylation of cysteine and variable oxidation of methionine and tryptophan to kynurenine or double oxidation to N′-Formylkynurenine. When a protein is identified as “hypothetical”, “unknown” or based on an expressed sequence tag (EST)-sequence, the sequence was used for a BLAST analysis, and the protein with the highest homology (when significant) was added as an identified protein.

All identifications were manually validated, and extra precursors were selected for fragmentation if the obtained data were judged as insufficient. When high quality spectra were not matched to sequences, a sequence was determined manually and/or the spectra were used for searches allowing for semi-tryptic peptides and common post-translational modifications (PTMs). This resulted in the identification of several signal cleavage sites that were confirmed either by homology to known signal cleavage sites or by using predictive software [92].

### 3.7. Carbohydrate Extraction and Quantification

Carbohydrates were extracted from samples that were composed of three to four shoots grown in the same culture container; five samples were taken per treatment and per species, amounting to five biological replicates. Each replicate was adjusted to about 100 mg of fresh matter and was ground in an Eppendorf tube of 2 mL (automatic grinder for 1 min at 22 Hz) with two metallic beads and 1 mL of a water/ethanol mixture (20/80, *v*/*v*). After vortexing and shaking for 30 min at 4 °C with an Eppendorf Thermomixer at 1400 rpm, samples were centrifuged at 17,000× *g* at 4 °C for 10 min. The supernatant was collected and the pellet extracted again with 0.5 mL water/ethanol (20/80, *v*/*v*). The resulting supernatant was pooled with the first one and dried at reduced pressure (Speedvac). The final dried extract was re-suspended in 1 mL Milli-Q water and filtered at 0.45 μm (PVDF filters) prior to analysis using High Performance Anion Exchange Chromatography coupled to a Pulsed Amperometric Detector (HPAEC-PAD) (Dionex ED 40, Dionex Corp., Sunnyvale, CA, USA), according to Guignard *et al.* [93]. Briefly, the analytical column was a Dionex Carbopac PA-20 (3 mm × 150 mm) with a PA-20 guard column (3 mm × 50 mm) kept at 35 °C. The mobile phase was on-line generated KOH at 0.5 mL min^−1^. The PAD detection was achieved with a gold working electrode and an Ag/AgCl reference electrode, with a data collection rate of 2 Hz. Carbohydrates were quantified using seven-points calibration curves with custom-made external standard solutions (based on stock solution of arabinose, galactose, glucose, sucrose, xylose, fructose, melibiose, raffinose, stachyose, maltose, cellobiose and rhamnose), ranging 1 to 100 μmol L^−1^, respectively, and every 10 injections, a check standard solution was used to confirm the calibration of the system.

The concentration of sugars was calculated based on the molar concentration obtained from the measurements and the initially used sample amount.

## 4. Conclusions

The initial growth inhibition under dehydration conditions occurs prior to any inhibition of photosynthesis or respiration [94]. Cell growth is among the primary processes to be affected by abiotic constraints, especially drought and cold. Growth is limited by the plant’s ability to osmotically adjust or conduct water, and this is normally done by the accumulation of osmotically active compounds. Soluble sugars act as potential signals, interacting with light, nitrogen and abiotic stress [95]. The accumulation of sugars under chilling temperatures follows the same trend in both species. Our results suggest an important role of sucrose, fructose and glucose in acclimation to cold. On the other hand, there is a larger carbohydrate accumulation in Désirée when submitted to osmotic stress. It suggests that the cleavage of sucrose might play a key function in Désirée under osmotic stress. Furthermore, our results are consistent, at least for Désirée, with the suggested relationship between the downregulation of ascorbate peroxidase, the downregulation of photosynthesis related proteins and lower growth.

One of the earliest responses to abiotic stresses and the inhibition of growth is the inhibition of protein synthesis [96]. Our results are consistent with the expected general changes under abiotic stress conditions. The inhibition of carbon metabolism and oxidative homeostasis appears to be a general response for all the stress treatments used and in both species. Furthermore, the differences between *S. commersonii* and Désirée are reflected in protein metabolism; the sucrose and sorbitol treatments induce more changes in *S. commersonii*, whereas cold affects more Désirée. Moreover, we found a lipid metabolism-related protein, patatin, that only increased in *S. commersonii* when treated with osmotic stress. This glycoprotein appears to have a role in Ca-dependent plant signal transduction [85]; our results are consistent with this concept, especially when considering the variation of differential abundant proteins related to protein metabolism in *S. commersonii* under osmotic stress conditions. They suggest that patatin might have a more important role in *S. commersonii* than in Désirée.

Sorbitol induces similar changes when compared to sucrose, and this is for both species. This suggests that both sugars could be comparable as stressors. However, we identified more stress-related proteins in Désirée under sorbitol treatment than in Désirée under sucrose treatment, specifically proteinase inhibitors. This suggests that both sugars induce some different pathways in Désirée, but no evidence was found.

In the case of cold, our data confirm the improved cold tolerance of *S. commersonii* and the higher chilling sensitivity of Désirée.

## Figures and Tables

**Table 1:**
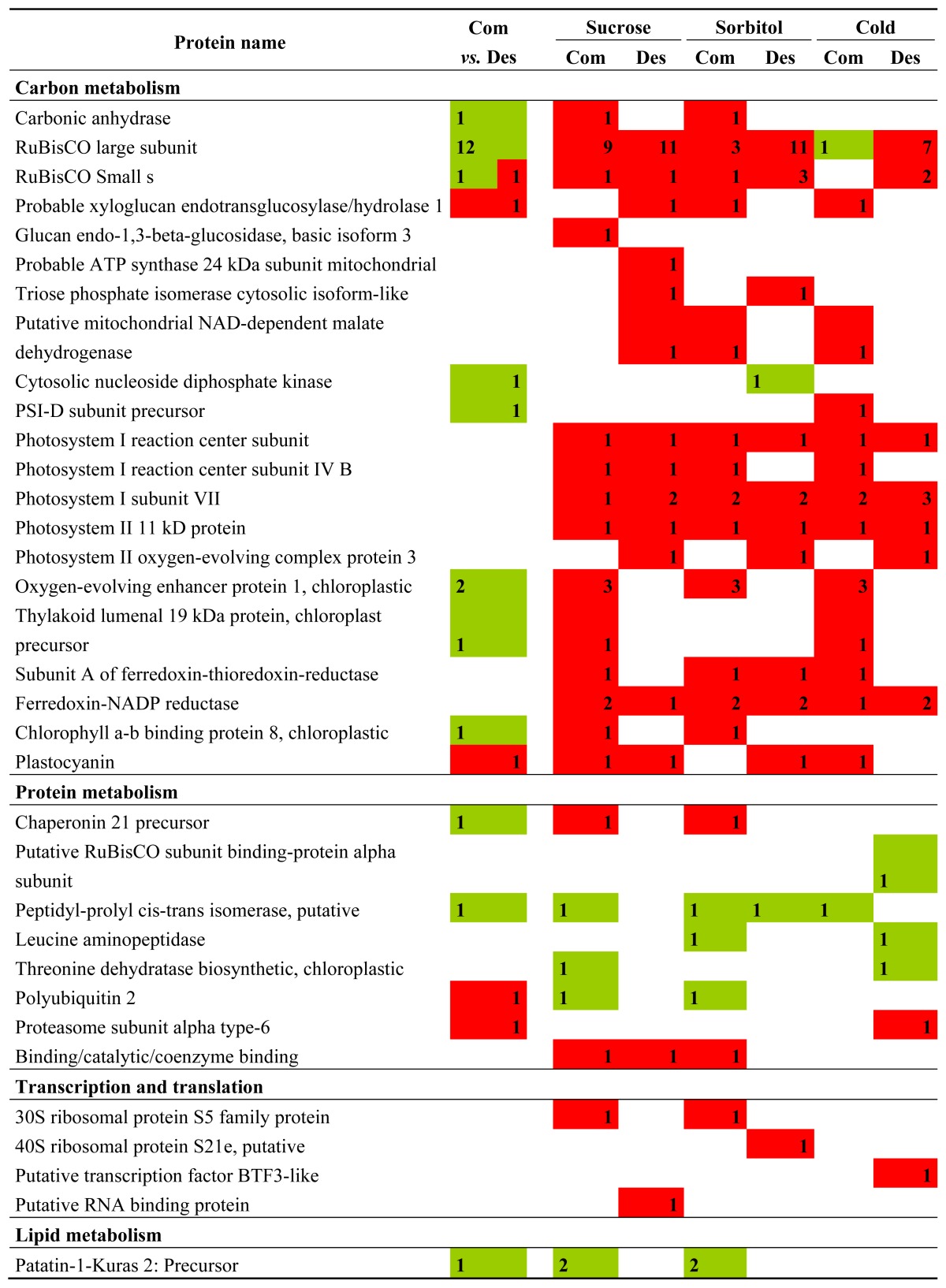

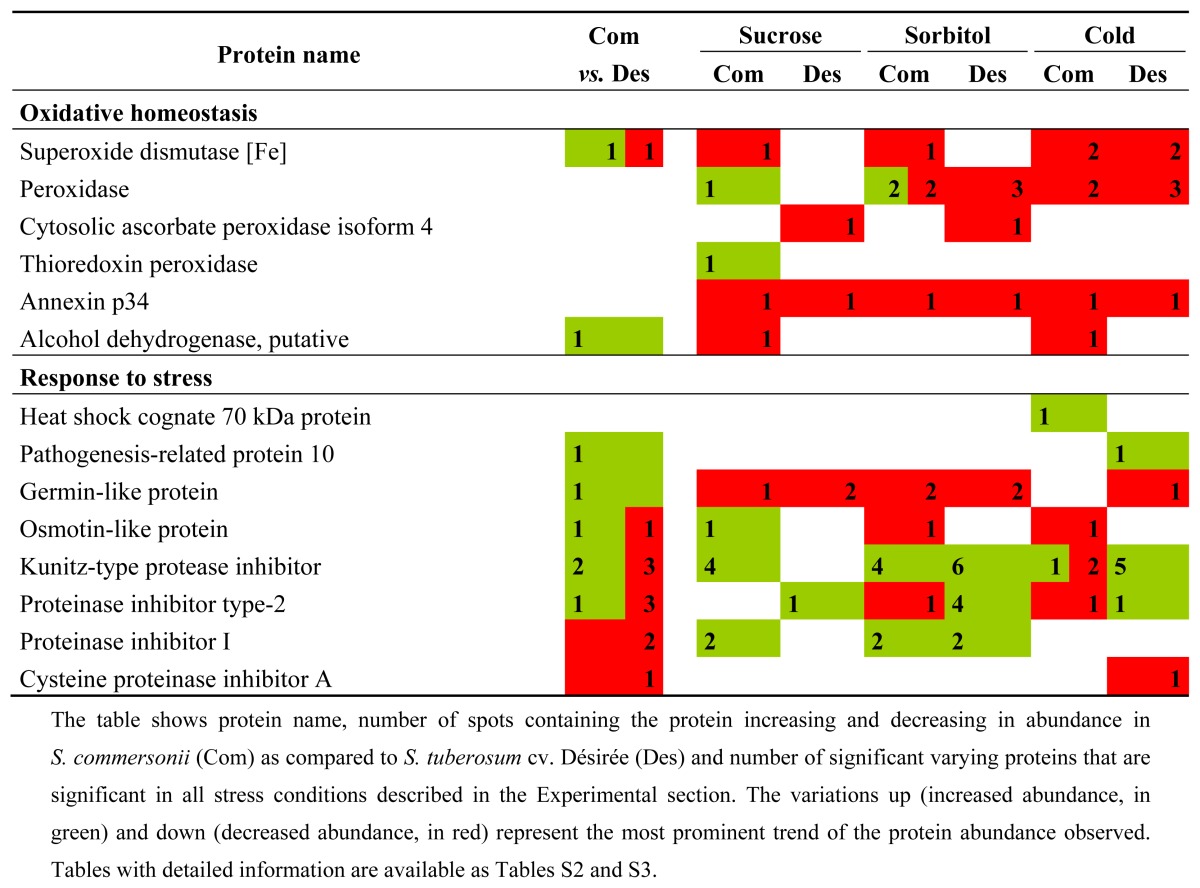
Summary of identified proteins in carbon metabolism, protein metabolism, transcription and translation, lipid metabolism, oxidative homeostasis and response to stress.

## Supplementary Files

Supplementary File 1 Supplementary Information (DOC, 96 KB)

Supplementary File 2 Supplementary Table 1 (XLSX, 27 KB)

Supplementary File 3 Supplementary Table 2 (XLS, 549 KB)
