# Supplementary material for: Differential Protein Expression in Response to Abiotic Stress in Two Potato Species: Solanum commersonii Dun and Solanum tuberosum L
Source: Int J Mol Sci. 2013 Mar 1;14(3):4912–33. doi: 10.3390/ijms14034912 (PMC3634427; doi:10.3390/ijms14034912)
Supplement: Supplementary File 1 — Supplementary Information (DOC, 96 KB) [file ijms-14-04912-s001.doc]

Supplementary Information

**Table S1.** Summary results of the analysis of carbohydrates. (Ctl, control; Suc, sucrose; Stl, Sorbitol) in *Solanum commersonii and Solanum* *tuberosum* L. cv. Désirée. Statistical significance was tested by one-way ANOVA between control and treated and between one variety against the other of the same condition. Letters represent statistical significance for ANOVA. *p* < 0.05. *n* = 5.

|  |  | *Solanum commersonii* (nmol/g FW) | | | | *Solanum tuberosum* L. cv. Désirée (nmol/g FW) | | | |
| --- | --- | --- | --- | --- | --- | --- | --- | --- | --- |
|  |  | Ctl | Suc | Stl | Cold | Ctl | Suc | Stl | Cold |
| Hexose | GALACTOSE | 178.86 ^b^ | 326.82 ^ab^ | 224.49 ^b^ | 268.88 ^b^ | 147.89 ^b^ | 492.92 ^a^ | 457.41 ^a^ | 609.21 ^a^ |
| Hexose | GLUCOSE | 15273.79 ^c^ | 23232.96 ^c^ | 18285.3 ^c^ | 40908.61 ^a^ | 3732.59 ^d^ | 22987.41 ^c^ | 16715.1 ^c^ | 31305.39 ^b^ |
| Hexose | FRUCTOSE | 16569.92 ^b^ | 15110.23 ^b^ | 15209.17 ^b^ | 26567.91 ^a^ | 4412.60 ^c^ | 9119.28 ^c^ | 8380.54 ^c^ | 18052.06 ^b^ |
| Disaccharide | SUCROSE | 8141.751 ^b^ | 14764.00 ^a^ | 9654.17 ^b^ | 15950.03 ^a^ | 3861.71 ^c^ | 5880.17 ^c^ | 5593.45 ^c^ | 10761.94 ^b^ |
| Disaccharide | MELIBIOSE (Gal-Glu) | 854.39 ^b^ | 802.77 ^b^ | 663.29 ^b^ | 1072.41 ^a^ | 409.60 ^c^ | 607.07 ^bc^ | 655.59 ^bc^ | 561.01 ^bc^ |
| Oligo | RAFFINOSE (Suc-Gal) | 1434.48 ^b^ | 1481.42 ^b^ | 1460.35 ^b^ | 1476.68 ^a^ | 1614.27 ^b^ | 1971.52 ^a^ | 1994.49 ^a^ | 1425.06 ^b^ |
| Oligo | STACHYOSE (Suc-Gal-Gal) | 644.13 ^d^ | 1688.98 ^b^ | 1113.89 ^c^ | 882.14 ^a^ | 637.57 ^d^ | 2160.50 ^c^ | 1039.43 ^c^ | 782.59 ^d^ |

**Table S4.** Proteinase inhibitors that appear in this study. The graph shows groups of different proteinase inhibitors after clustering and the table represent ratio (abundance of the proteins in the treated sample) of the identified proteins. A *t*-test was done and represented as *p-*value < 0.05. n.s, ratio value was not significant; Tables with detailed information are available as Supplemental data S1 and S2. Abbreviations: Com,
*S. commersonii*; Des, *S. tuberosum* L. cv. Désirée. gi, GenBank identifier.

**
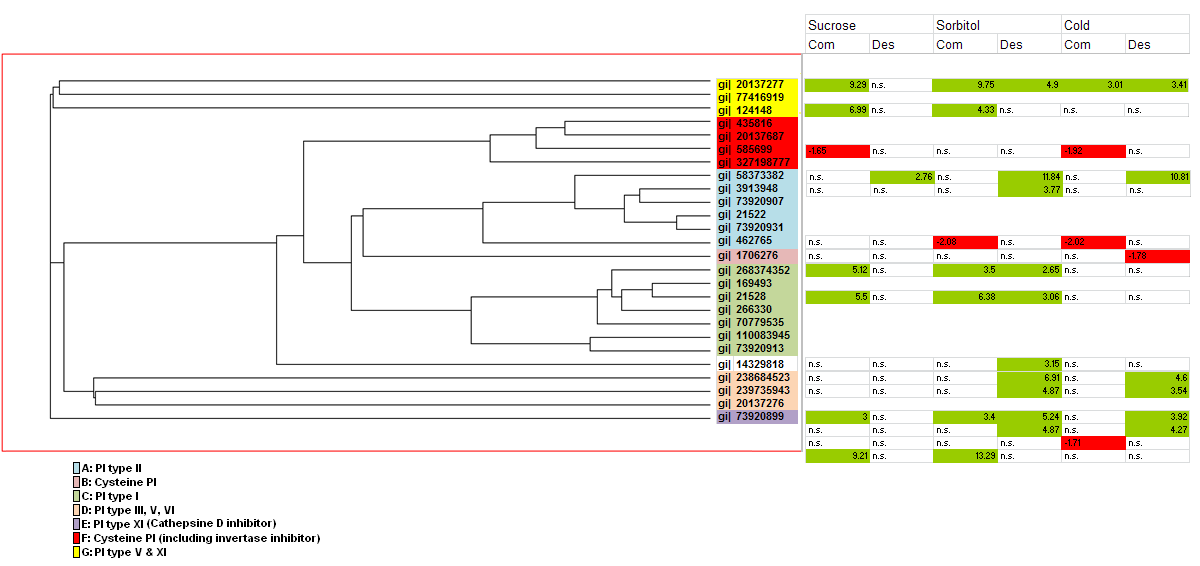
**

© 2013 by the authors; licensee MDPI, Basel, Switzerland. This article is an open access article distributed under the terms and conditions of the Creative Commons Attribution license (http://creativecommons.org/licenses/by/3.0/).
